# Standard (3, 5)-threshold quantum secret sharing by maximally entangled 6-qubit states

**DOI:** 10.1038/s41598-021-01893-0

**Published:** 2021-11-22

**Authors:** Yinxiang Long, Cai Zhang, Zhiwei Sun

**Affiliations:** 1 Department of Automation Engineering, Guangdong Technical College of Water Resources and Electric Engineering, Guangzhou, 510925 China; 2 College of Mathematics and Informatics, South China Agricultural University, Guangzhou, 510642 China; 3 School of Artificial Intelligence, Shenzhen PolyTechnic, Shenzhen, 518055 China

**Keywords:** Quantum information, Information theory and computation

## Abstract

In this paper, a standard (3, 5)-threshold quantum secret sharing scheme is presented, in which any three of five participants can resume cooperatively the classical secret from the dealer, but one or two shares contain absolutely no information about the secret. Our scheme can be fulfilled by using the singular properties of maximally entangled 6-qubit states found by Borras. We analyze the scheme’s security by several ways, for example, intercept-and-resend attack, entangle-and-measure attack, and so on. Compared with the other standard threshold quantum secret sharing schemes, our scheme needs neither to use *d*-level multipartite entangled states, nor to produce shares by classical secret splitting techniques, so it is feasible to be realized.

## Introduction

Classical secret sharing (CSS), proposed by Shamir^1^ and Blakley^2^ independently in 1979, is an important issue in modern cryptography. Its basic idea is to divide the classical secret into some shares such that the dealer can transmit the shares to the participants respectively through classical channel, and only all the participants work together can recover the secret, at the same time, some parts of them can not get any information of the secret. Hillery et al.^3^ proposed the concept of quantum secret sharing with reference to the classical secret sharing schemes, and designed two quantum secret sharing (QSS) schemes by using the quantum correlation of the GreenHorne–Zeilinger (GHZ) states in 1998. In Hillery’s QSS scheme of classical information, Alice shares her classical key with Bob and Charlie based on the correlations of the results of measurements with Pauli operators *X* or *Y*. However, in their QSS scheme of quantum information, teleportation is used for Alice to share quantum state with Bob and Charlie. In the same year, Karlsson et al.^4^ proposed another secret sharing protocol using the quantum correlation of two-particle entangled states, which encodes one bit information into unbiased orthogonal entangled state set $\{|\psi ^+\rangle ,|\phi ^-\rangle \}$ or $\{|\Psi ^+\rangle ,|\Phi ^-\rangle \}$ randomly in order to prevent the attack from a dishonest receiver or an eavesdropper, who wants to obtain the secret alone without the help of other agents by capturing both particles and performing a measurement with Bell basis. Here,

1 $$\begin{aligned} |\psi ^+\rangle= & {} \frac{1}{\sqrt{2}}(|01\rangle +|10\rangle ), |\phi ^-\rangle = \frac{1}{\sqrt{2}}(|00\rangle -|11\rangle ),\nonumber \\ |\Psi ^+\rangle= & {} \frac{1}{\sqrt{2}}(|\psi ^+\rangle +|\phi ^-\rangle ), |\Phi ^-\rangle = \frac{1}{\sqrt{2}}(|\psi ^+\rangle -|\phi ^-\rangle ). \end{aligned}$$

Guo^5^ proposed the first QSS scheme of classical key using product state as quantum channel. It is based on BB84 by encoding 1 bit with $\{|00\rangle ,|11\rangle \}$ or $\{|++\rangle ,|--\rangle \}$ randomly. Afterwards, several novel QSS protocols using single photon and local unitary operations were presented by Zhang^6^, Han^7^, and Yan^8^, respectively. Recently, Tavakoli^9^ pointed that a wide class of quantum protocols using *d*-level GHZ entanglement states (*d* is odd prime) can be mapped into simple ones involving one qudit, and proposed a QSS protocol which requires only sequential communication of a single *d*-level quantum system. Furthermore, Hao^10^ proposed a novel quantum secret sharing scheme by a single-particle $p^2$-dimensional quantum system (*p* is a prime) and unitary transformation between these mutually unbiased bases.

However, entangled states play a more important role in all sorts of quantum information processing tasks including QSS. Up to now, in addition to Hillery’s and Karlsson’s schemes mentioned earlier, a large number of QSS schemes have been proposed based on various entangled states, such as GHZ states^11–14^, GHZ like states^15,16^, Bell states^17–21^, genuine multiparty entangled states (including maximally genuine multiparty entangled states, cluster states and graph states, etc.)^22–26^, and *d*-level entangled states (for example, *d*-level GHZ states and *d*-level Bell states)^27–32^.

A $(t, n)(t\le n)$ threshold secret sharing (abbreviated to TSS) scheme divides a secret into *n* pieces such that any *t* or more than *t* out of *n* pieces can recover the secret, while less than *t* pieces can not. Shamir and Blakley proposed independently the first threshold classical secret sharing (abbreviated to TCSS) schemes, which are called Lagrange interpolating polynomial scheme and vector scheme, respectively. Naturally, if a TSS scheme is implemented by quantum technique, it is called (*t*, *n*) threshold quantum secret sharing (abbreviated to TQSS) scheme.

By using the technique of quantum error correction coding, the first TQSS scheme sharing quantum states was proposed by Cleve^33^ in 1999. In 2013, Gheorghiu^34^ introduced a scheme by embedding a classical linear code into a quantum error-correcting code and then mapping the latter to a quantum secret sharing protocol. In this protocol, some of the players are only required to perform local measurements and share their measurement results via classical channels.

It is worth pointing out that TQSS schemes based on quantum error correction coding can also be used to share classical messages because schemes sharing quantum state can also share classical information. However, these methods are not easy to be implemented because they usually require complicated operations and *d*-level multiparty entangled states.

Another importatnt idea of TQSS sharing classical information is to employ Shamir’s secret splitting technique^1^ to produce shares (classical information) and transmit shares by quantum mechanics. At present, a large number of TQSS schemes sharing classical information have been developed. Some examples are listed as follows:

1. In 2005, Tokunaga^35^ presented the notion of threshold collaborative unitary transformation or threshold quantum cryptography. It employs Shamir’s secret splitting technique and avoids the constraint of the quantum no-cloning theorem.
2. In 2008, Yang^36^ proposed a (*t*, *m*)-(*s*, *n*) TQSS scheme, in which any *t* of *m* members in group 1 can recover the secret in cooperation with any *s* of *n* members in group 2 using a sequence of single photons. The president firstly generates a classical key *K* and randomly divides it into *K*1 and *K*2 whose values meet $K=K_{1}\bigoplus K_{2}$. He makes *m*(*n*) shares of the *K*1(*K*2) using Shamir’s secret splitting technique.
3. In 2009, Li^37^ proposed a TQSS scheme to share classical secret based on Bell states and Pauli operators by using Shamir’s secret splitting technique.
4. In 2013, Massoud^38^ proposed a (*t*, *m*)-(*s*, *n*) TQSS scheme to share classical secret based on GHZ states and Pauli operators by using Shamir’s secret splitting technique.
5. In 2015, Qin^39^ proposed a TQSS scheme to share a quantum state based on the phase shift operation on single qubit by using Shamir’s secret splitting technique, in which the participants perform the phase shift operations on the quantum state according to their private keys, and any *t* out of the *n* participants can reconstruct the original quantum state. In 2018, Lu^40^ proposed another TQSS scheme to share classical information in addition to quantum states by using the similar idea to Qin’s.
6. In 2016, Qin^41^ proposed a TQSS scheme based on single particle by using Shamir’s secret splitting technique, in which the Hash function is used to guarantee the security of particle transmission.
7. In 2017, Song^42^ proposed a TQSS scheme sharing a *d*-dimensional classical secret based on quantum Fourier transformation (QFT) and a *d*-level GHZ state by using Shamir’s secret splitting technique. However, Kao^43^ points out a calculation problem in Song’s paper, which indicates that the agents are unable to obtain the boss’s secret information. But, they have not suggested any improvement of the scheme in Ref.^42^ to mitigate this loophole. To mitigate the loophole, Roy^44^ has recently proposed a TQSS scheme sharing a *d*-level classical message based on a *d*-dimensional multi-particle entangled state by using QFT and classical secret splitting technique.
8. In 2019, Bai^45^ proposed a quantum secret sharing scheme using a set of orthogonal generalized Bell states in $C^4\bigotimes C^4$ and local distinguishability. In their proposed protocol the participants use one-way loop classical communication and local projective measurements to distinguish between the orthogonal states. And combined with the classical Shamir (*t*, *n*)-threshold scheme, a (*t*, *n*)-threshold quantum scheme was presented.

It is worth pointing out that TQSS schemes based on classical secret splitting technique are easy to understand, but they are not purely quantum and they are complex in calculating the shares.

In 2015, Rahaman^14^ presented a novel restricted (2, *n*)-threshold LOCC-QSS scheme, in which any two cooperating players, one from each of two disjoint groups of players, can always reconstruct the secret based on the local distinguishability of *n*-qubit GHZ states. So far, based on the local distinguishability of multiparty entangled states, a great deal of threshold LOCC-QSS schemes have been found successively and summarized below:

1. In 2015, Yang^46^ presented a standard (2, *n*)-threshold LOCC-QSS scheme, in which any two players can collaboratively recover the secret, using some pairs of locally distinguishable orthogonal *d*-level multipartite entangled states to represent the encoded secret.
2. In 2017, Bai^28^ proposed a standard (2, *n*)-threshold LOCC-QSS scheme and a restricted (2, *n*)-threshold LOCC-QSS scheme based on the local distinguishability of an orthogonal pair of *n*-qudit GHZ states.
3. In 2017, using the discriminability of two orthogonal *d*-level GHZ states under LOCC, Bai^47^ proposed multiple QSS schemes to realize three types of access structures, i.e., the (*n*, *n*)-threshold, the restricted (3, *n*)-threshold and restricted (4, *n*)-threshold.
4. In 2017, Wang^48^ proposed the concept of judgment space to investigate the quantum secret sharing scheme based on local distinguishability, and developed a standard (3, 4)-threshold LOCC-QSS scheme and a standard (5, 6)-threshold LOCC-QSS scheme with three orthogonal 4-qudit (4-level) entangled states and three orthogonal 6-qudit (6-level) entangled states, respectively. Furthermore, Liu^49^ proposed a standard (6, 7)-threshold LOCC-QSS scheme with five orthogonal 7-qudit (7-level).
5. In 2020, Dou^50^ followed the work of Wang^48^ and investigate the judgement space deeply. The digital representation and graphical representation of judgement space were given, and an algorithm was designed to search optional states for any given *k* and *n*.
6. In 2018, Bai^51^ constructed a group of orthogonal multipartite entangled states in *d*-dimensional system and investigated the distinguishability of these entangled states under restricted local operations and classical communications, and proposed a restricted (5, *n*)-threshold quantum secret sharing scheme and a restricted (5, 8)-threshold quantum secret sharing scheme as an example based on these properties.

It is worth pointing out that, in the TQSS schemes based on the local distinguishability of multiple orthogonal entangled quantum states, we need to use high-dimensional quantum systems, namely qudit states instead of qubits.

From above, it is known that complicated entangled qudit states or classical secret splitting technique is required in the existing standard TQSS schemes. However, TQSS schemes that are based on qubit system and do not require classical secret splitting technique are easier to implement. So, we study this question and propose a novel standard TQSS scheme sharing classical secret, in which the maximally entanged 6-qubit state (for convenience, called it BPB state) discovered by Borras^52^ is used as channel and no classical secret splitting technique is required.

The remainder of this paper is organized as follows. Firstly, some singular properties of the BPB state will be described, and a standard (3, 5)-TQSS protocol sharing classical messages by the BPB states is presented. Then, the security of the protocol is analyzed. Finally, we conclude with a summary.

## Standard (3, 5)-threshold quantum secret sharing

In this section, a standard (3, 5)-threshold quantum secret sharing scheme based on the BPB states is presented. In our protocol, we adopt the data block transmission technique^53^ and the decoy photon technique^24,54–56^ to assure the security of the transmission. First some key properties of the BPB state are developed, then a standard (3, 5)-threshold quantum secret sharing scheme is constructed by using the singular properties of the BPB state and Bell states, and finally the security analysis of the proposed protocol is presented.

### The properties of the BPB state

The BPB state discovered by Borras is in the following form:

2 $$\begin{aligned}&\frac{1}{\sqrt{32}}\bigg [\Big (|000000\rangle +|111111\rangle +|000011\rangle +|111100\rangle \nonumber \\&\quad +|000101\rangle +|111010\rangle +|000110\rangle +|111001\rangle \nonumber \\&\quad +|001001\rangle +|110110\rangle +|001111\rangle +|110000\rangle \nonumber \\&\quad +|010001\rangle +|101110\rangle +|010010\rangle +|101101\rangle \nonumber \\&\quad +|011000\rangle +|100111\rangle +|011101\rangle +|100010\rangle \Big )\nonumber \\&\quad -\Big (|010100\rangle +|101011\rangle +|010111\rangle +|101000\rangle \nonumber \\&\quad +|011011\rangle +|100100\rangle +|001010\rangle +|110101\rangle \nonumber \\&\quad +|001100\rangle +|110011\rangle +|011110\rangle +|100001\rangle \Big )\bigg ]_{123456}. \end{aligned}$$

Hereafter, subscripts $\{1,2,3,4,5,6\}$ represent the serial number of particles.

Now, let us rewrite the 6-qubit entangled state in the following form of generalized Schmidt decomposition of three-partite split (12|36|45):

3 $$\begin{aligned} 1/2\bigg (|\phi ^+\rangle _{12}|\phi ^+\rangle _{36}|\phi ^+\rangle _{45}+|\phi ^-\rangle _{12}|\psi ^-\rangle _{36}|\psi ^+\rangle _{45}\nonumber \\ +|\psi ^-\rangle _{12}|\psi ^+\rangle _{36}|\phi ^-\rangle _{45}+|\psi ^+\rangle _{12}|\phi ^-\rangle _{36}|\psi ^-\rangle _{45}\bigg ). \end{aligned}$$

By formula (3), we have:

4 $$\begin{aligned} \rho _1= & {} \rho _2=\rho _3=\rho _4=\rho _5=\rho _6=\frac{1}{2}I_2, \end{aligned}$$

5 $$\begin{aligned} \rho _{12}= & {} \rho _{36}=\rho _{45}=\frac{1}{4}I_4, \end{aligned}$$

6 $$\begin{aligned} \rho _{123}= & {} \rho _{124}=\rho _{125}=\rho _{126}=\frac{1}{8}I_8,\nonumber \\ \rho _{361}= & {} \rho _{362}=\rho _{364}=\rho _{365}=\frac{1}{8}I_8,\nonumber \\ \rho _{451}= & {} \rho _{452}=\rho _{453}=\rho _{456}=\frac{1}{8}I_8. \end{aligned}$$

Hereafter, $\rho _{i},\rho _{ij}$ and $\rho _{ijk}$ represent reduced density operators of particles $\{i\}$, $\{i,j\}$ and $\{i,j,k\}$, respectively, and $I_2,I_4$ and $I_8$ represent the identity density operators on two-dimensional Hilbert space $H_2$, four-dimensional Hilbert space $H_2^{\otimes 2}$ and eight-dimensional Hilbert space $H_2^{\otimes 3}$, respectively.

Similarly, we can reformulate $|\Psi \rangle _{6qb}$ in the forms of generalized Schmidt decomposition of three-partite split (13|24|56),(14|26|35),(15|23|46) and (16|25|34), respectively as follows:

7 $$\begin{aligned}&1/2\bigg (-|\phi ^-\rangle _{13}|\phi ^-\rangle _{24}|\phi ^+\rangle _{56}+|\phi ^+\rangle _{13}|\psi ^+\rangle _{24}|\psi ^+\rangle _{56}\nonumber \\&\quad -|\psi ^+\rangle _{13}|\psi ^-\rangle _{24}|\phi ^-\rangle _{56}-|\psi ^-\rangle _{13}|\phi ^+\rangle _{24}|\psi ^-\rangle _{56}\bigg ), \end{aligned}$$

8 $$\begin{aligned}&1/2\bigg (|\phi ^-\rangle _{14}|\phi ^+\rangle _{26}|\phi ^-\rangle _{35}+|\phi ^+\rangle _{14}|\psi ^+\rangle _{26}|\psi ^+\rangle _{35}\nonumber \\&\quad +|\psi ^-\rangle _{14}|\psi ^-\rangle _{26}|\phi ^+\rangle _{35}+|\psi ^+\rangle _{14}|\phi ^-\rangle _{26}|\psi ^-\rangle _{35}\bigg ), \end{aligned}$$

9 $$\begin{aligned}&1/2\bigg (|\phi ^+\rangle _{15}|\phi ^+\rangle _{23}|\phi ^+\rangle _{46}+|\phi ^-\rangle _{15}|\psi ^+\rangle _{23}|\psi ^-\rangle _{46}\nonumber \\&\quad +|\psi ^+\rangle _{15}|\psi ^-\rangle _{23}|\phi ^-\rangle _{46}+|\psi ^-\rangle _{15}|\phi ^-\rangle _{23}|\psi ^+\rangle _{46}\bigg ), \end{aligned}$$

10 $$\begin{aligned}&1/2\bigg (|\phi ^-\rangle _{16}|\phi ^+\rangle _{25}|\phi ^-\rangle _{34}+|\phi ^+\rangle _{16}|\psi ^-\rangle _{25}|\psi ^-\rangle _{34}\nonumber \\&\quad +|\psi ^+\rangle _{16}|\psi ^+\rangle _{25}|\phi ^+\rangle _{34}+|\psi ^-\rangle _{16}|\phi ^-\rangle _{25}|\psi ^+\rangle _{34}\bigg ). \end{aligned}$$

In the same way, by formulas (7)–(10), we have:

11 $$\begin{aligned}&\rho _{13}=\rho _{24}=\rho _{56}=\frac{1}{4}I_4,\nonumber \\&\rho _{14}=\rho _{26}=\rho _{35}=\frac{1}{4}I_4,\nonumber \\&\rho _{15}=\rho _{23}=\rho _{46}=\frac{1}{4}I_4,\nonumber \\&\rho _{16}=\rho _{25}=\rho _{34}=\frac{1}{4}I_4, \end{aligned}$$

12 $$\begin{aligned}&\rho _{134}=\rho _{135}=\rho _{146}=\rho _{156}=\frac{1}{8}I_8,\nonumber \\&\rho _{234}=\rho _{235}=\rho _{246}=\rho _{256}=\frac{1}{8}I_8. \end{aligned}$$

According to formula (4), it is known that there is 1 ebit of entanglement between any one particle and the other particles. Similarly, we have, there exists 2 ebits of entanglement between any splits of $(2-particles|4-particles)$ by formulas (5) and (11), and 3 ebits of entanglement between any splits of $(3-particles|3-particles)$ by formulas (6) and (12).

From formulas (3) and (7)–(10), it is known that the other four particles will collapsed to tensor product of two pairs of EPR when we measure the BPB state on any two qubits $\{i,j\}$ with the Bell states basis $\{|\phi ^+\rangle ,|\phi ^-\rangle , |\psi ^+\rangle ,|\psi ^-\rangle \}$. For example, suppose that the result of measurement on particles 1 and 2 is $|\phi ^+\rangle$, then the other four particles will collapse to $|\phi ^+\rangle _{36}|\phi ^+\rangle _{45}$ (see Table 1).

**Table 1:** The other four qubits will collapse to tensor product of two pairs of EPR when we measure the BPB state on any two qubits *i*, *j* (for example, i=1) with the Bell basis.

*i*, *j*	Measure results on particles *i*, *j*	Corresponding states of the other four particles	*i*, *j*	Measure results on particles *i*, *j*	Corresponding states of the other four particles
1,2	$|\phi ^+\rangle$	$|\phi ^+\rangle _{36}|\phi ^+\rangle _{45}$	1,2	$|\psi ^+\rangle$	$|\phi ^-\rangle _{36}|\psi ^-\rangle _{45}$
1,2	$|\phi ^-\rangle$	$|\psi ^-\rangle _{36}|\psi ^+\rangle _{45}$	1,2	$|\psi ^-\rangle$	$|\psi ^+\rangle _{36}|\phi ^-\rangle _{45}$
1,3	$|\phi ^+\rangle$	$|\psi ^+\rangle _{24}|\psi ^+\rangle _{56}$	1,3	$|\psi ^+\rangle$	$|\psi ^-\rangle _{24}|\phi ^-\rangle _{56}$
1,3	$|\phi ^-\rangle$	$|\phi ^-\rangle _{24}|\phi ^+\rangle _{56}$	1,3	$|\psi ^-\rangle$	$|\phi ^+\rangle _{24}|\psi ^-\rangle _{56}$
1,4	$|\phi ^+\rangle$	$|\psi ^+\rangle _{26}|\psi ^+\rangle _{35}$	1,4	$|\psi ^+\rangle$	$|\phi ^-\rangle _{26}|\psi ^-\rangle _{35}$
1,4	$|\phi ^-\rangle$	$|\phi ^+\rangle _{26}|\phi ^-\rangle _{35}$	1,4	$|\psi ^-\rangle$	$|\psi ^-\rangle _{26}|\phi ^+\rangle _{35}$
1,5	$|\phi ^+\rangle$	$|\phi ^+\rangle _{23}|\phi ^+\rangle _{46}$	1,5	$|\psi ^+\rangle$	$|\psi ^-\rangle _{23}|\phi ^-\rangle _{46}$
1,5	$|\phi ^-\rangle$	$|\psi ^+\rangle _{23}|\psi ^-\rangle _{46}$	1,5	$|\psi ^-\rangle$	$|\phi ^-\rangle _{23}|\psi ^+\rangle _{46}$
1,6	$|\phi ^+\rangle$	$|\psi ^-\rangle _{25}|\psi ^-\rangle _{34}$	1,6	$|\psi ^+\rangle$	$|\psi ^+\rangle _{25}|\phi ^+\rangle _{34}$
1,6	$|\phi ^-\rangle$	$|\phi ^+\rangle _{25}|\phi ^-\rangle _{34}$	1,6	$|\psi ^-\rangle$	$|\phi ^-\rangle _{25}|\psi ^+\rangle _{34}$

It is necessary to point out the form and properties of the Bell states. There are four orthogonal Bell states as follows:

13 $$\begin{aligned} |\phi ^+\rangle= & {} \frac{1}{\sqrt{2}}(|00\rangle +|11\rangle ) = \frac{1}{\sqrt{2}}(|++\rangle +|--\rangle )\nonumber \\ |\phi ^-\rangle= & {} \frac{1}{\sqrt{2}}(|00\rangle -|11\rangle ) =\frac{1}{\sqrt{2}}(|+-\rangle +|-+\rangle )\nonumber \\ |\psi ^+\rangle= & {} \frac{1}{\sqrt{2}}(|10\rangle +|01\rangle ) = \frac{1}{\sqrt{2}}(|++\rangle -|--\rangle )\nonumber \\ |\psi ^-\rangle= & {} \frac{1}{\sqrt{2}}(|10\rangle -|01\rangle ) = \frac{1}{\sqrt{2}}(|+-\rangle -|-+\rangle ) \end{aligned}$$

Here, $|+\rangle =\frac{1}{\sqrt{2}}(|0\rangle +|1\rangle ), |-\rangle =\frac{1}{\sqrt{2}}(|0\rangle -|1\rangle )$. It is known to all that, for any Bell state, if we apply a local unitary operator from $\{\sigma _x,\sigma _y,\sigma _z\}$ on any particle of the Bell state, then it will be transformed into another orthogonal Bell state (see Table 2). That is to say, $|\phi ^+\rangle$ ($|\psi ^-\rangle$) will be transformed into $|\psi ^-\rangle$ ($|\phi ^+\rangle$ ) if we apply a unitary operation $I\otimes \sigma _y$ or $\sigma _y\otimes I$ to it. From formula (13), we have that the two Bell states $\{|\phi ^+\rangle ,|\psi ^-\rangle \}$ can be distinguished by local measure with basis $\{|0\rangle ,|1\rangle \}$ or $\{|+\rangle ,|-\rangle \}$, so can the two Bell states $\{|\phi ^-\rangle ,|\psi ^+\rangle \}$. For example, for given two Bell states $|\phi ^+\rangle$ and $|\psi ^-\rangle$, if the result of measurement is $|0\rangle |0\rangle$ ($|+\rangle |+\rangle$) or $|1\rangle |1\rangle$ ($|-\rangle |-\rangle$), then we can conclude that the Bell state is $|\phi ^+\rangle$.

**Table 2:** An arbitrary Bell state will transform to another Bell state if a Pauli operator is applied on any one of its particles.

	Bell state $|\phi ^+\rangle$	Bell state $|\phi ^-\rangle$	Bell state $|\psi ^+\rangle$	Bell state $|\psi ^-\rangle$
$I\otimes I$	$|\phi ^+\rangle$	$|\phi ^-\rangle$	$|\psi ^+\rangle$	$|\psi ^-\rangle$
$I\otimes \sigma _x$ or $\sigma _x\otimes I$	$|\psi ^+\rangle$	$|\psi ^-\rangle$	$|\phi ^+\rangle$	$|\phi ^-\rangle$
$I\otimes \sigma _y$ or $\sigma _y\otimes I$	$|\psi ^-\rangle$	$|\psi ^+\rangle$	$|\phi ^-\rangle$	$|\phi ^+\rangle$
$I\otimes \sigma _z$ or $\sigma _z\otimes I$	$|\phi ^-\rangle$	$|\phi ^+\rangle$	$|\psi ^-\rangle$	$|\psi ^+\rangle$

### Standard (3, 5)-threshold quantum secret sharing scheme of classical message

Now, let us construct the standard (3, 5)-threshold quantum secret sharing scheme based on the BPB state by the correlations of the measurement results on it and local distinguishability of Bell states. Our scheme is divided into four phases: preparing phase, checking phase, coding phase and decoding phase.

1. Phase for preparing BPB states and inserting decoy photons. In this step, the dealer Alice prepares *N* BPB states indexed from 1 to *N*. All particles numbered $i(1\le i\le 6)$ in the BPB states constitute a particle sequence $S_i$ with length *N*. Then, Alice prepares randomly a different sequence, $r_i=\Pi _i(1,2,\ldots ,N)$, for each user Bob$_i$. Here, $\Pi _i(1,2,\ldots ,N)$ represents an arbitrary permutation of the sequence $(1,2,\ldots ,N)$. Now, for each particle sequence $S_i(i=1,2,\ldots ,6)$, Alice exchanges the order of particles in it to make a new sequence $S_i^{'}$ according to the permutation sequence $r_i$. In order to detect eavesdropping, for each particle sequence $S_i^{'}$, Alice prepares some decoy particles, which are randomly in the states $\{|0\rangle ,|1\rangle ,|+\rangle ,|-\rangle \}$, and randomly inserts these decoy particles into the sequence $S_i^{'}(1\le i\le 6)$ to make a new sequence $S_i^{''}(1\le i\le 6)$ with length $N^{'}$. Finally, She keeps a record of the position and the initial state of each decoy particle, and sends the $i-{th}(i(1\le i\le 6))$ sequence $S_i^{''}$ to the $i-{th}$ agent Bob$_i$ who saves these particles in quantum register to be used in the future. After receiving all the $N^{'}$ particles, Bob$_i$($1\le i \le 6$) announces the fact.
2. Phase for checking eavesdropping by decoy photons. After confirming that Bob$_i$ ($1\le i \le 6$) has received the sequence $S_i^{''}$, Alice publicly announces the position of the decoy particles via classical channel and asks Bob$_i (1\le i\le 6)$ to measure these particles with the basis $\{|0\rangle ,|1\rangle \}$ or $\{|+\rangle ,|-\rangle \}$ chosen randomly. Bob$_i (1\le i\le 6)$ publishes his measurement base and results. For the decoy particles measured with correct base, Alice can compute the error rate by comparing the measurement results to the initial states. If the error rate exceeds the predefined threshold value, Alice aborts the process and starts a new one because quantum communication between Alice and the agents may be attacked. Otherwise, they continue the protocol.
3. Phase for sharing secret. After Alice has distributed *N* BPB states to Bob$_i$ ($1\le i \le 6$) in security, she informs each Bob$_i$($1\le i \le 6$) of the order of the particles sent to him, respectively, i.e., the permutation $\Pi _i(1,2,\ldots ,N)$. Then Bob$_i$ reorders his particles by the permutation. Now, any one user from $\{$ Bob$_1$,Bob$_2$,Bob$_3$,Bob$_4$,Bob$_5$,Bob$_6\}$ can share *N* bits of message among the other five users using the *N* BPB states. That is to say, each BPB state can be used to share one bit. Suppose Bob$_k$ $(1\le k \le 6)$ wants to share *N* bits $\{b_0b_1\cdots b_N\}$ among the other five users. Now, Bob$_k$ performs an appropriate unitary operation $U_j$ on the $j-{th}$ particle to encode the secret $b_j$. Here, $U_j$ satisfy:
  $$\begin{aligned} U_j=\left\{ \begin{aligned} I&,&b_j=0 \\ \sigma _y&,&b_j=1 \\ \end{aligned} \right. \end{aligned}$$
  Then, $Bob_k$ measures each of his own particles with basis $\{|0\rangle ,|1\rangle \}$ or $\{|+\rangle ,|-\rangle \}$ randomly, respectively. Finally, $Bob_k$ publishes the measurements through classical channel.
4. Phase for recovering the secret messages. After $Bob_k$ has shared secret messages, any three of the other five users can work together to recover the secrets to be shared. Let’s give an example to illustrate it. Suppose $Bob_1$ has shared his secrets by the procedure above. Now, any three users from $\{Bob_j| 2\le j\le 6\}$ can unite to disclose the secrets by the following method:

Two users can make joint measurements on their particles in the same BPB state with Bell basis, and the third user makes local measurement with the same basis as $Bob_1$. Now, By combining their own measurements with $Bob_1$’s measurements, they can obtain the secrets. For an instance (see Table 3), suppose that $Bob_2,Bob_3$ and $Bob_4$ want to recover the secret shared by $Bob_1$, then $Bob_2$ and $Bob_4$ can unite to make Bell measurement, and $Bob_3$ can make local measurement using the same basis as $Bob_1$. Further, suppose that the joint measurement result from $Bob_2$ and $Bob_4$ is $|\phi ^+\rangle$, and the results from $Bob_3$ and $Bob_1$ are $|+\rangle$ and $|+\rangle$, respectively, then we can infer that the secret is 1. It is worth pointing out, to fulfil the task, only specific two users(e.g., $Bob_2$ and $Bob_4$ in the example above), instead of any two users, can be chosen to unite to make measurement with Bell basis. By formulas (3) and (7)–(10), we can know which two of the three users need to make joint measurement with Bell basis, and which one needs to make local measurement in order to recover the secrets (see Table 4).

**Table 3:** $Bob_2$,$Bob_3$ and $Bob_4$ can unite to recover the secret shared by $Bob_1$ through combining the *Z*(*X*) basis measurement by $Bob_1$, the *Z*(*X*) basis measurement by $Bob_3$ and the joint measurements by $Bob_2$ and $Bob_4$.

Measurement results with Bell basis by $Bob_2$ and $Bob_4$	Measurement results with *Z*(*X*) basis by $Bob_3$	Measurement results with *Z*(*X*) basis by $Bob_1$	Operators for encoding	Secrets to be recovered
$|\phi ^+\rangle$	$|0\rangle (|+\rangle )$	$|1\rangle (|-\rangle )$	*I*	0
$|\phi ^+\rangle$	$|1\rangle (|-\rangle )$	$|0\rangle (|+\rangle )$	*I*	0
$|\phi ^+\rangle$	$|0\rangle (|+\rangle )$	$|0\rangle (|+\rangle )$	$\sigma _y$	1
$|\phi ^+\rangle$	$|1\rangle (|-\rangle )$	$|1\rangle (|-\rangle )$	$\sigma _y$	1
$|\phi ^-\rangle$	$|0\rangle (|+\rangle )$	$|0\rangle (|-\rangle )$	*I*	0
$|\phi ^-\rangle$	$|1\rangle (|-\rangle )$	$|1\rangle (|+\rangle )$	*I*	0
$|\phi ^-\rangle$	$|0\rangle (|+\rangle )$	$|1\rangle (|+\rangle )$	$\sigma _y$	1
$|\phi ^-\rangle$	$|1\rangle (|-\rangle )$	$|0\rangle (|-\rangle )$	$\sigma _y$	1
$|\psi ^+\rangle$	$|0\rangle (|+\rangle )$	$|0\rangle (|+\rangle )$	*I*	0
$|\psi ^+\rangle$	$|1\rangle (|-\rangle )$	$|1\rangle (|-\rangle )$	*I*	0
$|\psi ^+\rangle$	$|0\rangle (|+\rangle )$	$|1\rangle (|-\rangle )$	$\sigma _y$	1
$|\psi ^+\rangle$	$|1\rangle (|-\rangle )$	$|0\rangle (|+\rangle )$	$\sigma _y$	1
$|\psi ^-\rangle$	$|0\rangle (|+\rangle )$	$|1\rangle (|+\rangle )$	*I*	0
$|\psi ^-\rangle$	$|1\rangle (|-\rangle )$	$|0\rangle (|-\rangle )$	*I*	0
$|\psi ^-\rangle$	$|0\rangle (|+\rangle )$	$|0\rangle (|-\rangle )$	$\sigma _y$	1
$|\psi ^-\rangle$	$|1\rangle (|-\rangle )$	$|1\rangle (|+\rangle )$	$\sigma _y$	1

**Table 4:** Any three users from $\{Bob_2,Bob_3,Bob_4,Bob_5,Bob_6\}$ can unite to recover the secret shared by Bob1 through choosing two users to make Bell measurement and the third to make local measurement.

Participating players	Players to make unite measurement	Player to make local measurement
$Bob_2,Bob_3,Bob_4$	$Bob_2,Bob_4$	$Bob_3$
$Bob_2,Bob_3,Bob_5$	$Bob_2,Bob_3$	$Bob_5$
$Bob_2,Bob_3,Bob_6$	$Bob_3,Bob_6$	$Bob_2$
$Bob_2,Bob_4,Bob_5$	$Bob_4,Bob_5$	$Bob_2$
$Bob_2,Bob_4,Bob_6$	$Bob_2,Bob_6$	$Bob_4$
$Bob_2,Bob_5,Bob_6$	$Bob_2,Bob_5$	$Bob_6$
$Bob_3,Bob_4,Bob_5$	$Bob_3,Bob_5$	$Bob_4$
$Bob_3,Bob_4,Bob_6$	$Bob_3,Bob_4$	$Bob_6$
$Bob_3,Bob_5,Bob_6$	$Bob_5,Bob_6$	$Bob_3$
$Bob_4,Bob_5,Bob_6$	$Bob_4,Bob_6$	$Bob_5$

## Security analysis of the presented TQSS protocols

In this section, we’ll discuss how this protocol can prevent a dishonest Bob or a third eavesdropper Eve from acquiring the secret without being detected. In general, there are usual two types of attack method from attackers, i.e., intercept and resend attack, and entanglement attack.

In our protocol, the decoy photons widely adopted in quantum secret sharing are used to check eavesdropping^39–41^. Consequently, when eavesdropper Eve intends to excute intercept-and-resend attack and tries to obtain the transmitted message, he can only intercept the quantum sequence but can not acquire the sequence states, and thus fails to resend a perfect copy of the sequence due to Heisenberg uncertainty principle and quantum no-cloning principle. Because Eve does not know the positions and states of the decoy photons, the attack will cause an increase in error rate to about 1/2 and thus be detected. On the other hand, If Eve possesses several agents’ particles, he might attack successfully by making union measurement on his particles with Bell basis. However, since Eve can not distinguish which particles contribute to the same BPB state in the preparing phase, his measure must bring error rate of 1/2 in checking phase.

By the same analysis as Jiang^24^ and Wang^56^, entangle-and-measure attack will cause error and be detected in checking step due to the decoy photons. In other words, if Eve wanted to get some useful information by entangling the attached particles, he will inevitably introduce interference, which will be found by the participants in the eavesdropping detection.

## Conclusion

We have presented a standard (3, 5)-threshold quantum secret sharing protocol by genuinely maximally entangled 6-qubit states, i.e., BPB states. In our protocol, six users $\{Bob_1,Bob_2,Bob_3,Bob_4,Bob_5,Bob_6\}$ a priori share a series of BPB states, of which $Bob_i$ owns the $i-{th}$ particle of each BPB state. A BPB state can be reformulated as generalized Schimdt decomposition of any split $(ij|kl|mn)(1\le i\ne j\ne k\ne l\ne m\ne n \le 6)$, and the results of measurement on partial particles of the BPB state are correlative. Based on these singular properties, any three users except $Bob_i$ can recover the secret shared by $Bob_i$. In fact, after preparing step, any one of the six users can share secret, and any three from the other five users can unite to recover the secret. The presented protocol is secure against eavesdropping by inserting the decoy photons into the distributed particle sequences.

A brief comparison of the various existing TQSS schemes sharing classical secrets with our scheme is summarized in Table 5. In summary, our protocol has the advantage that, it uses qubit entangled state instead of *d*-level entangled state as channel, in addtion it doesn’t need to utilize Shamir’s classical threshold scheme to produce secret shares. Therefore, our method is more simple and feasible.

**Table 5:** A brief comparison of the various existing standard TQSS schemes sharing classical information (for convenience, we divide them into three classes) with our scheme.

	The first class	The second class	The third class	Our scheme
Represntative	Cleve^33^	Tokunaga^35^	Yang^46^	Our scheme
Existing protocols	^33,34^	^35,38–42,44,45^	^46,48–50^	Our scheme
Main princple	Based on quantum error correction coding	Based on classical secret splitting techique and QKD	Based on local distinguishability of specific *d*-level entangled states	Based on singular propertites of the maximally qubit entangled states
Quantum channels	*d*-Level entangled state	Single particle qubit or qudit entangled state	Specfic *d*-level entangled state	The maximally entangled 6-qubit state
Quantum computation	Unitary operations on multi-particles	Unitary operation on single particle, e.g. $\sigma _x$, QFT, etc	Local projective measurements	Measurements with *X*(*Z*) basis or Bell basis
Classical computation	No need	Need	No need	No need
